# Jellyfish mucin may have potential disease-modifying effects on osteoarthritis

**DOI:** 10.1186/1472-6750-9-98

**Published:** 2009-12-08

**Authors:** Naoshi Ohta, Masato Sato, Kiminori Ushida, Mami Kokubo, Takayuki Baba, Kayoko Taniguchi, Makoto Urai, Koji Kihira, Joji Mochida

**Affiliations:** 1 Department of Orthopaedic Surgery, Surgical Science, Tokai University School of Medicine, 143 Shimokasuya, Isehara, Kanagawa 259-1193, Japan; 2 Eco-Soft Materials Research Unit, Advanced Research Institute, Riken, 2-1 Hirosawa, Wako, Saitama 351-0198, Japan; 3 Jellyfish Research Laboratories, Inc, KSP E513 Sakado 3-2-1, Takatsu-ku, Kawasaki, Kanagawa 213-0012, Japan

## Abstract

**Background:**

We aimed to study the effects of intra-articular injection of jellyfish mucin (qniumucin) on articular cartilage degeneration in a model of osteoarthritis (OA) created in rabbit knees by resection of the anterior cruciate ligament. Qniumucin was extracted from *Aurelia aurita *(moon jellyfish) and *Stomolophus nomurai *(Nomura's jellyfish) and purified by ion exchange chromatography. The OA model used 36 knees in 18 Japanese white rabbits. Purified qniumucin extracts from *S. nomurai *or *A. aurita *were used at 1 mg/ml. Rabbits were divided into four groups: a control (C) group injected with saline; a hyaluronic acid (HA)-only group (H group); two qniumucin-only groups (M groups); and two qniumucin + HA groups (MH groups). One milligram of each solution was injected intra-articularly once a week for 5 consecutive weeks, starting from 4 weeks after surgery. Ten weeks after surgery, the articular cartilage was evaluated macroscopically and histologically.

**Results:**

In the C and M groups, macroscopic cartilage defects extended to the subchondral bone medially and laterally. When the H and both MH groups were compared, only minor cartilage degeneration was observed in groups treated with qniumucin in contrast to the group without qniumucin. Histologically, densely safranin-O-stained cartilage layers were observed in the H and two MH groups, but cartilage was strongly maintained in both MH groups.

**Conclusion:**

At the concentrations of qniumucin used in this study, injection together with HA inhibited articular cartilage degeneration in this model of OA.

## Background

Osteoarthritis (OA) is one of the most common joint diseases and is characterized by the gradual degeneration of cartilage over a long time (regressive degeneration). This disease commonly develops in the weight-bearing joints of the lower limbs, such as the knee and hip joints, and onset shows a close correlation closely with age. OA is thus one of the main causes of pain and joint dysfunction among the elderly, and is also often seen in young people after traumas such as a fracture, anterior cruciate ligament transection (ACL-T), meniscus injury or in the presence of an underlying disease such as hemophilia [1]. Currently, pharmacotherapies for OA focus mainly on the alleviation of pain and consist of systemic analgesic therapies and local intra-articular treatments. Nonsteroidal anti-inflammatory drugs (NSAIDs) are widely used as systemic analgesic therapies [2]. However, pathological progression of OA can be accelerated by the use of NSAIDs [3-6]. Similarly, hyaluronan (HA) injection therapy is a widely recognized part of local intra-articular treatments, inhibiting the destruction of articular cartilage by increasing the viscosity of synovial fluid [7-10].

The presence of a thin membrane layer on the articular cartilage surface is believed to protect against external impact and reduce friction. This membrane is formed from glycoproteins with a mucin-type domain, some of which have been identified in humans, including tribonectin and lubricin [11,12]. These glycoproteins in the joints show tandem repeat regions composed of 7-8 amino acids in the mucin domain; more than 90% of the threonines and serines can form *O*-glycosyl bonds and are glycosylated. The sugar chains are short, consisting of 2-3 monosaccharides (including sialic acid), with very little diversity (glycoforms) in the sugar chains. These mucins display characteristics very similar to those of qniumucin, the jellyfish mucin analyzed in this study.

At present, no methods have been established to produce mucins artificially on a sufficiently large scale for therapeutic use. Chemical synthesis is not very practical, as the cost of producing a mucin-type polymer is high, even when the structure is very simple. Although *O*-glycosylation is a typical posttranslational modification in biological systems, occurring in the Golgi apparatus, synthetic versions of this procedure, in which sugar chains are attached after the expression of core proteins, can only be performed in a limited manner. Under these circumstances, extraction of natural abundant mucins from organisms is most often used. In the industrial production of mucins, only extraction from the gastric juices or saliva of domestic animals has proven commercially successful. However, the purity and homogeneity of these mucins are insufficient for use as a single substance and they have been further avoided since the discovery of bovine spongiform encephalopathy (BSE) [13]. Mucins have also been extracted from marine creatures, such as starfish [14] and squid [15], but this technique is also ineffective in terms of efficiency and cost.

**Figure 1:**
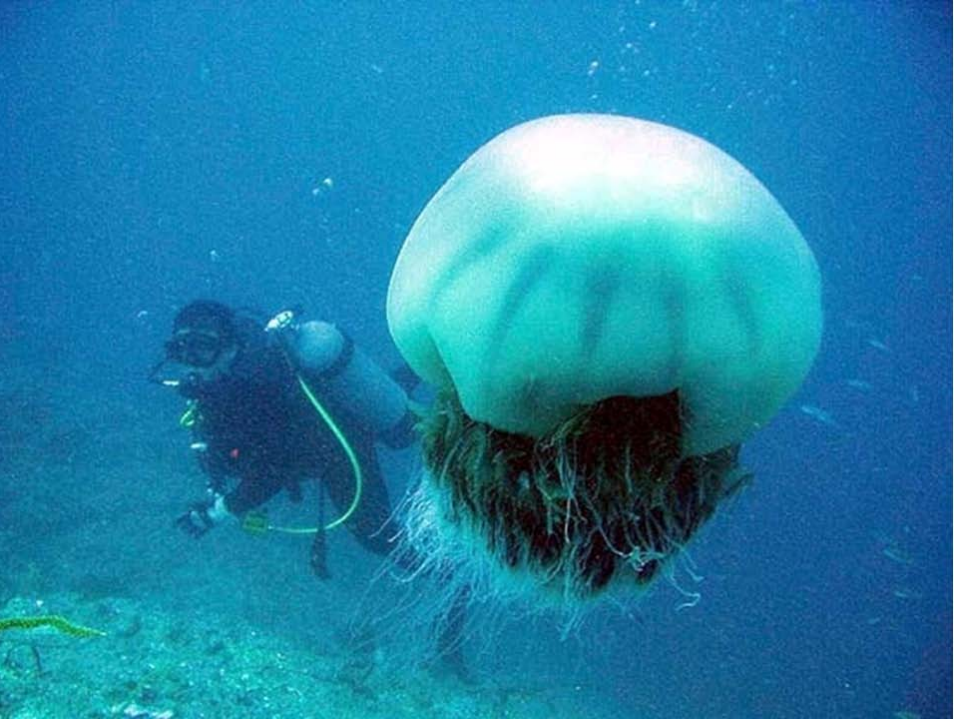
**Nomura's jellyfish**. The world's largest jellyfish, growing to over 1 m in diameter and weighing more than 200 kg.

*Stomolophus nomurai *is the world's largest jellyfish, growing to over 1 m in diameter and weighing more than 200 kg (Fig. 1). Vast proliferation of this species has been reported in the coastal regions of the Sea of Japan. Massive smacks of jellyfish (about 100 tons on 1 day at one site) are caught in fishing nets, significantly interfering with the fishing industry. Removal of these jellyfish has now become a routine practice for power plants, industrial facilities, fisheries and harbors in coastal areas, and handling such large quantities of jellyfish is extremely difficult. On the positive side, Ushida *et al. *have discovered and successfully isolated a novel mucin derived from many species of jellyfish, including *S. nomurai *[16]. This compound, qniumucin, is low in diversity and high in purity and constitutes an exceptional mucin that can be obtained industrially as a homogeneous product.

In this study, we discuss the effects of intra-articular injection of qniumucin on cartilage degeneration in a rabbit model of OA.

## Results

### Macroscopic study

**Figure 2:**
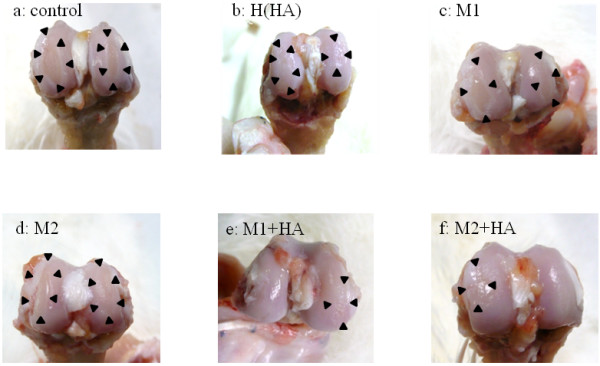
**Macroscopic findings in the femur of the knee joint 10 weeks after OA model preparation**. **a **(group C): Cartilage defects are observed on both the medial and lateral sides. These extend to the subchondral bone and range across the entire weight-bearing area. **b **(group H): Irregularity of the cartilage surface is apparent on both medial and lateral sides in the photo. A cartilage defect extending to the subchondral bone can be seen in some sections. **c, d **(group M1, moon jellyfish extract; group M2, Nomura's jellyfish extract, respectively): As with group C, a cartilage defect extending to the subchondral bone is observed. No difference is apparent in the range of the defect. **e, f **(group MH1, moon jellyfish extract; group MH2, Nomura's jellyfish extract, respectively): Irregularity of the cartilage surface is apparent on the medial side in the photo. No exposure of the subchondral bone is observed and the range of the defect is very limited. No differences are seen in the M or MH groups where the qniumucin had been extracted from the two different species of jellyfish. Arrowheads indicate areas of exposed subchondral bone.

Advanced cartilage defects extending to the subchondral bone were observed on both the medial and lateral sides in group C (Fig. 2a). Similar results were observed in group M, with no apparent differences between groups C and M (Fig. 2a, c, d). In a comparison of groups H and MH, less cartilage degeneration was observed in the groups treated with qniumucin and HA than in the group treated with HA alone (Fig. 2b, e, f).

### Histological study

**Figure 3:**
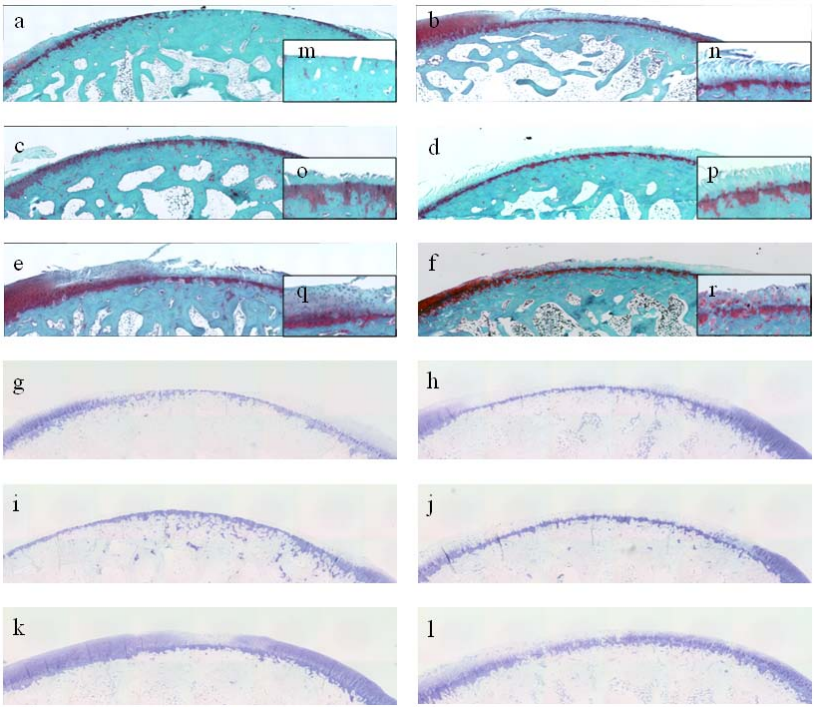
**Histological findings**. The femoral condyle in the knee joint was resected and fixed in 4% neutral-buffered formalin (pH 7.4). The tissue was then decalcified with 10% EDTA and the cross-section was embedded in paraffin wax. Dewaxed sections were processed for safranin-O (a--f) and toluidine blue (g--l) staining. **a **(group C): The cartilage defect reaches the subchondral bone. **b **(group H): The fissure in the cartilage extends to the deep layer and reduced staining is observed in the upper layer from the tide mark. Cell sequences are not maintained and a reduction in the number of cells is observed. **c, d **(group M1, moon jellyfish extract; group M2, Nomura's jellyfish extract, respectively): Only a small portion of the cartilage layer remains and the cells have been obliterated. **e, f **(group MH1, moon jellyfish extract; group MH2, Nomura's jellyfish extract, respectively): Peeled cartilage layers are seen, but fissure have generally extended to the middle layer. Cell sequences are relatively well maintained and the reduction in staining is minor compared with that in group H. **g **(group C): No metachromasia is observed with toluidine blue staining in the cartilage layer. **h **(group H): Slight metachromasia is observed in the cartilage layer. **i, j **(group M1, moon jellyfish extract; group M2, Nomura's jellyfish extract, respectively): No metachromasia is observed in the cartilage layer. **k, l **(group MH1, moon jellyfish extract; group MH2, Nomura's jellyfish extract, respectively): The cartilage layer shows metachromasia with toluidine blue staining, which is specific for articular cartilage. Magnification: a-l, × 4; m-r, × 10.

Exposure of subchondral bone was detected by safranin-O staining in group C (Fig. 3a). No cartilaginous layer with metachromasia shown by toluidine blue staining was observed in group C (Fig. 3g). Fissures in the cartilage layers and reduced stainability were observed in group H (Fig. 3b). Slight metachromasia in the cartilage layer was observed in group H (Fig. 3h). In group M, minimal residual cartilage layers were present, but no staining was observed and the cartilage cells had been destroyed (Fig. 3c, d). As in group C, no metachromatic cartilage layer was observed in group M (Fig. 3i, j). Erosion of the cartilage, fissures and reduced staining were observed in group MH. However, compared with group H, the fissures were shallower and more cells were present (Fig. 3e, f). No metachromasia was observed in the cartilage layer in group MH (Fig. 3k, l).

**Figure 4:**
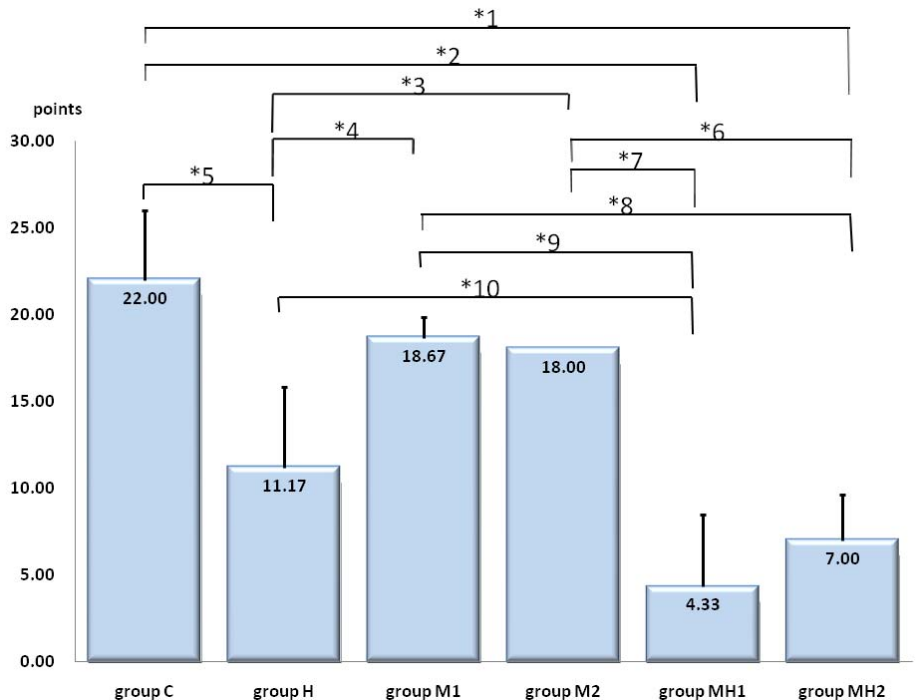
**Osteoarthritis score**. Samples were histologically evaluated based on the standard grading and staging of OA cartilage histopathology. Evaluation was quantified using the following formula: most degenerated site in the cartilage (grades 1-6, Table 2) × area of degeneration (stages 1-4, Table 3) = OA score (1-24, Table 3). Columns and vertical lines represent mean values and standard deviations for each group, respectively. No significant differences were observed between group C and groups M1 and M2. Significant differences were observed between groups H and C, between groups H and M1 or M2, between groups H and MH1, and between groups M1 or M2 and groups MH1 or MH2. *Significant differences between groups, corresponding to the last column in Table 1. After analysis of variance, least significant differences were used for post hoc testing (Scheffé's method). Mean OA scores were compared and values of * *P *< 0.05 were considered significant. Groups M1 and MH1: moon jellyfish extract. Groups M2 and MH2: Nomura's jellyfish extract.

Average OA scores were: group C, 22.00 ± 4.00; group H, 11.17 ± 4.65; group M1, 18.67 ± 1.15; group M2, 18.00 ± 0.00; group MH1, 4.33 ± 4.16; and group MH2, 7.00 ± 2.65. Groups C, M1 and M2 did not differ significantly. Significant differences were observed between groups H and C, between groups H and M1 or M2, between groups H and MH1, and between groups M1 or M2 and groups MH1 or MH2 (*P < 0.05) (Fig. 4, Table 1).

**Table 1:** Results of post hoc testing (Scheffé's method).

(I) V1	(J) V1	Difference of averages (I-J)	SEM	P	95% CI		
						
					Lower limit	Upper limit	Figure 5
Group C	group MH1	17.6667*	2.3094	.000	11.891	23.443	*2
	
	group MH2	15.0000*	2.3094	.000	9.224	20.776	*1
	
	group H	10.8333*	2.3094	.002	5.057	16.609	*5
	
	group M1	3.3333	2.3094	.233	-2.443	9.109	
	
	group M2	4.0000	2.3094	.157	-1.776	9.776	

Group H	group MH1	6.8333*	2.6822	.024	1.057	12.609	*10
	
	group MH2	4.1667	2.6822	.142	-1.609	9.943	
	
	group C	-10.8333*	2.6822	.002	-16.609	-5.057	*5
	
	group M1	-7.5000*	2.6822	.015	-13.276	-1.724	*4
	
	group M2	-6.8333*	2.6822	.024	-12.609	-1.057	*3

Group M1	group MH1	14.3333*	0.6667	.000	8.557	20.109	*9
	
	group MH2	11.6667*	0.6667	.001	5.891	17.443	*8
	
	group H	7.5000*	0.6667	.015	1.724	13.276	*4
	
	group C	-3.3333	0.6667	.233	-9.109	2.443	
	
	group M2	.6667	0.6667	.806	-5.109	6.443	

Group M2	group MH1	13.6667*	0.0000	.000	7.891	19.443	*7
	
	group MH2	11.0000*	0.0000	.001	5.224	16.776	*6
	
	group H	6.8333*	0.0000	.024	1.057	12.609	*3
	
	group C	-4.0000	0.0000	.157	-9.776	1.776	
	
	group M1	-.6667	0.0000	.806	-6.443	5.109	

Group	group MH2	-2.6667	2.4037	.334	-8.443	3.109	
	
	group H	-6.8333*	2.4037	.024	-12.609	-1.057	*10
	
	group C	-17.6667*	2.4037	.000	-23.443	-11.891	*2
	
	group M1	-14.3333*	2.4037	.000	-20.109	-8.557	*9
	
	group M2	-13.6667*	2.4037	.000	-19.443	-7.891	*7

Group MH2	group MH1	2.6667	1.5275	.334	-3.109	8.443	
	
	group H	-4.1667	1.5275	.142	-9.943	1.609	
	
	group C	-15.0000*	1.5275	.000	-20.776	-9.224	*1
	
	group M1	-11.6667*	1.5275	.001	-17.443	-5.891	*8
	
	group M2	-11.0000*	1.5275	.001	-16.776	-5.224	*6

SEM, standard error of the mean; CI, confidence interval.

## Discussion

This study examined the effects of intra-articular injection of qniumucin in a rabbit model of OA using resection of the anterior cruciate ligament. The results were as follows. First, when qniumucin alone was injected, cartilage degeneration did not differ from that in the control group. Second, only minor cartilage degeneration was observed when a mixture of HA and qniumucin was injected compared with the degeneration present after injection of HA only. Third, no significant difference in the effects of qniumucin isolated from different species of jellyfish was apparent in this study.

HA is responsible for the viscosity and elasticity of synovial fluid and thus plays roles in lubrication and shock-absorption. An increase in low molecular weight HA reportedly reduces the viscosity and elasticity of synovial fluid under inflammatory conditions such as OA [17]. However, various other effects have been attributed to HA, including anti-inflammatory and analgesic effects, inhibition of cartilage degeneration and an ability to enhance damage repair [18].

Safranin-O staining reflects the accumulation of proteoglycan and acts as an index of cartilage degeneration in tissues. Although a reduction in safranin-O staining of the extracellular matrix was seen in our study, cartilage degeneration was clearly inhibited in groups H and MH compared with the degeneration observed in group C.

In recent years, a glycoprotein with a mucin region called lubricin (a superficial zone protein and member of the tribonectin family) has been identified in the synovial fluid and on the articular surface. The presence of lubricin in both areas contributes to reductions in articular friction. This substance is characterized by a mucin-type region in which *O*-glycans are connected to the protein backbone, with nonmucin-type sequences at both ends. This nonmucin region has been suggested to interact with the cartilage surface to facilitate the adherence of lubricin. The mucin and nonmucin regions are assumed to play different roles, with the former extending the sugar chains outward like a brush to reduce friction and the latter promoting adhesion of lubricin to the cartilage surface. This is referred to as the "brushing model", based on the inferred shape of the molecule [19]. No such mechanism has been suggested for tribonectin, but this substance shares some common characteristics with lubricin insofar as it also displays a mucin-type sequence that reduces friction together with a nonmucin region. The mucin region is believed to adsorb densely to the cartilage tissue surface (perhaps as a film) to reduce friction. However, adhesion of these mucin-type glycoproteins to the articular surface after injection has yet to be directly confirmed.

Mucin or mucin-like substances are likely to form a self-assembled film (SAF) on both hydrophobic and hydrophilic surfaces. One significant example of a SAF in biological systems is the mucin film on the ocular surface that protects the eyeball [20]. Although the bond created by each single sugar chain makes only a small contribution to the total adsorption energy, cooperative interaction of many glycan chains concentrated in a small area provides a sufficient gain in free energy (and a reduction in entropy) to immobilize the polymer chain of the mucin. Adhesion and SAF formation on the surface are possible without any selective interaction, such as that suggested for the nonmucin sequence of lubricin, which may be the first trigger of adhesion. Therefore, if the goal of treatment is to reduce friction, any kind of mucin that lacks a nonmucin region could be used instead of lubricin or tribonectin. Jay mentioned the synergic effects of lubricin and HA in an in vitro study [12]. Mucin may have potential synergic effects with HA, as mucin is one component of lubricin. In this study, we first demonstrated that exogenous mucin derived from natural jellyfish showed synergic effects with HA using an in vivo animal model. These effects might be induced by improving the viscosity and friction properties of synovial fluid and enhancing the self-assembly capacity of cartilage.

Many different kinds of mucins are known. As no large-scale production of artificial polymeric mucins has been achieved with biotechnology or chemical synthesis, extraction from the natural environment remains the most appropriate method for provision as commercial substances. Mucins are widely distributed in animals and plants as components of mucus. For example, in plants mucins are found in extracts of lotuses, okra and yams. However, the structure of plant mucins is completely different from that of animal mucins, with a short peptide connected to long sugar chains, such as galactan and mannan [21]. We have therefore focused on animal mucins as candidate materials for therapeutic injection.

Animal mucins are produced and retained as components of mucus by all living animals, regardless of taxonomy. Huge potential variations exist in components such as the core peptide sequence, nonmucin domain sequence and structure of the sugar chains (glycoforms) according to the animal species. However, there are very few examples of animal mucins that are mass produced by domestic animals [13]. These are broadly classified as gastric mucins [22] and submaxillary salivary gland mucins [23,24]. Gastric mucins constitute a mixture harvested from the lavage fluids of internal organs as low-purity materials. Only total monosaccharide (for example, sialic acid) and amino acid analyses have been performed, with no further characterization, so gastric mucins constitute an inexpensive material suitable for mass production. In contrast, submaxillary mucins are very pure and detailed structural analyses of their amino acid sequences and saccharide compositions have been performed [25]. A monoclonal antibody directed against submaxillary mucins known as sialyl Tn antigen has been produced and used as a tumor marker [26]. However, these animal mucins risk contamination with foreign substances unless thorough purification is performed, so use tends to be avoided. For example, prions causing BSE cannot be eliminated. Mucins from snails [27], starfish [14] and squid [15] are currently available on the market, but production volumes are limited. Among these mucin alternatives, qniumucin, which is harvested from jellyfish, was discovered in recent years by our colleagues [16]. As mass production is inexpensive, qniumucin is a candidate mucin for wide application in many patients as a treatment for OA. Qniumucin is characteristically an almost pure monotonously repeated sequence of short mucin regions called "tandem repeats". The tandem repeat unit consists of eight amino acids (sequence VVETTAAP or VIETTAAP) and the sugar chains are short (usually only 1-3 sugars), with only a few types of monosaccharides and no sialic acid. Since qniumucin seems to have very few peptide sequences other than the mucin portion, only mild biological reactions are expected from the immune system in the form of allergies. This low potential for biological rejection is a further advantage of the use of this mucin. The risks involved in providing a mass product on an industrial scale are thus significantly reduced.

In a preliminary experiment to test the effects of injection with qniumucin, no elevation of the blood cell count or C-reactive protein and no swelling of the joints were observed (unpublished results). No histological findings of synovium have been detected among normal and injected joints (unpublished results). The possibility that an endogenous endotoxin, identified in this preliminary experiment, might cause adverse effects is a concern, but no such effects have yet been observed. After the purification method was improved to preclude any contamination with the solvent from high-performance liquid chromatography, the concentration of endotoxin in qniumucin purified by ion-exchange chromatography and in unpurified qniumucin were both <10 EU/ml when characterized by Endosafe-PTS (Charles River Laboratories Japan, Kanagawa, Japan). Based on these considerations, we proceeded with the intra-articular injection of this qniumucin, expecting a friction-reducing effect. The present results represent a promising step in the development of a new treatment for cartilage degeneration.

## Conclusion

After injecting a mixture of mucin and HA, cartilage degeneration was significantly inhibited compared with that in rabbits injected with HA alone. This effect might be induced by improving the viscosity and friction properties of the synovial fluid and enhancing the self-assembly capacity of the cartilage.

## Methods

All procedures using animals in this study were performed in accordance with the Guide for the Care and Use of Laboratory Animals (NIH Publication No. 85-23, revised 1996) published by the National Institutes of Health, USA, and the Guidelines of Tokai University on Animal Use.

### Reagents

Artz^® ^(Kaken Pharmaceutical, Tokyo, Japan) was used in making the solution of polymeric HA. The average molecular weight of this HA is approximately 800,000 and the concentration used was 25 mg/2.5 ml. Qniumucin from *S. nomurai *or *A. aurita *was dissolved in saline at a concentration of 1 mg/ml to create stock solutions.

### Extraction and purification of qniumucin

The mesogloea, the major part of the umbrella in jellyfish, was cut into small pieces and suspended in water. After removing insoluble material by centrifugation at 10,000 × *g*, one third of the volume of ethanol was added to the supernatant. The resulting precipitate was harvested by centrifugation at 10,000 × *g *and dissolved in water. Supernatant was collected by centrifugation at 10,000 × *g*, dialyzed against water, and lyophilized. The lyophilized material was then dissolved in phosphate buffer and incubated with anion-exchange gel beads (Diethylaminoethyl (DEAE) - resin, Toyopearl DEAE-650M; Tosoh, Tokyo, Japan) for 1 h. The beads were washed well with phosphate buffer and the bound proteins were eluted with elution buffer (phosphate buffer, 0.5 M NaCl). The eluent was collected by filtration, dialyzed against water, and lyophilized.

### Animals

Japanese white rabbits (females weighing 3 kg) were purchased from Tokyo Laboratory Animals Science (Tokyo, Japan). The rabbits were kept individually and reared in a fiber-reinforced polymer cage (width 450 mm × height 450 mm × depth 900 mm).

### OA model

An ACL-T model [28-34] was prepared for use as the OA model. With the rabbits under inhalation anesthesia with isoflurane (Forane^®^; Abbott Japan, Tokyo, Japan), a 3-cm incision was made aseptically on the medial side of both knees to expose the patella and patellar tendon, and the articular capsule was incised. The patella was then dislocated outwardly to an extended position and the knee joint was bent for macroscopic resection of the anterior cruciate ligament. The patella was then repositioned and the subdermal muscular layer and skin were sutured with nylon thread.

### Experimental design

**Figure 5:**
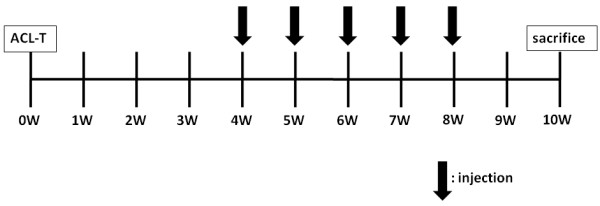
**Protocol**. Intra-articular injections were commenced from week 4 after preparation of the osteoarthritis (OA) model. Treatment solutions were injected once weekly until week 8. The rabbits were killed 10 weeks after surgery. Surgery and intra-articular injections were performed with the rabbits under general anesthesia. ACL-T, anterior cruciate ligament transection.

Eighteen rabbits in which the 36 anterior cruciate ligaments had been resected in both knee joints were divided randomly into four groups: a control (C) group (injected with saline; six knees), an HA-only group (H group; six knees), two qniumucin-only groups (M1:*S. nomurai*, M2:*A. aurita *group; six knees each) and two qniumucin + HA groups (MH1 and MH2 groups; six knees each). Each treatment comprised 1 ml per intra-articular injection. Purified qniumucin from *S. nomurai *or *A. aurita *was used at 1 mg/ml. Mixtures of HA solution (0.5 ml) + saline (0.5 ml), qniumucin solution (0.5 ml) + saline (0.5 ml) and qniumucin solution (0.5 ml) + HA solution (0.5 ml) were injected into the H, M and MH groups, respectively, after resection of the anterior cruciate ligament. These four treatments were given at intervals of 7 days, starting from week 4 after surgery (thus in weeks 4, 5, 6, 7 and 8; Fig. 5). With each rabbit under isoflurane inhalation anesthesia, the intra-articular injection was made into the upper margin of the lateral patella in the rabbit knee joint, using a syringe with a 26-G needle (Terumo, Tokyo, Japan). All rabbits were killed by pentobarbiturate overdose (Nembutal^®^; Dainippon Sumitomo Pharma, Osaka, Japan) in week 10 after resection (Fig. 5).

### Macroscopic and histological evaluation of the articular cartilage

The femoral knee joint was checked immediately after the rabbits had been killed. The femora of both knee joints were resected and fixed in 4% neutral-buffered formalin (pH 7.4). Fixed samples were decalcified with 10% ethylene diaminetetraacetic acid (EDTA) (pH 7.4) and then embedded in paraffin wax. Sections cut in the sagittal plane of the femoral condyle were dewaxed, prepared and stained with safranin-O and toluidine blue. Samples were evaluated histologically based on the standard grading and staging of OA cartilage histopathology [35]. Evaluation was quantified with the following formula: most degenerated site in the cartilage (grades 1-6, Table 2) × the area of degeneration (stage 1-4, Table 3) = OA score (1-24, Table 3).

**Table 2:** OA cartilage histopathology grade assessment; grading methodology

Grade (key feature)	Associated criteria (tissue reaction
Grade 1: surface intact	Matrix: superficial zone intact, oedema and/or superficial fibrillation (abrasion), focal superficial matrix condensation Cells: death, proliferation (clusters), hypertrophy, superficial zone Reaction must be more than superficial fibrillation only
	
Grade 2: surface discontinuity	As above + Matrix discontinuity at superficial zone (deep fibrillation) ± Cationic stain matrix depletion (Safranin O or Toluidine Blue) upper 1/3 of cartilage ± Focal perichondronal increased stain (mid zone) ± Disorientation of chondron columns Cells: death, proliferation (clusters), hypertrophy
	
Grade 3: vertical fissures (clefts)	As above Matrix vertical fissures into mid zone, branched fissures ± Cationic stain depletion (Safranin O or Toluidine Blue) into lower 2/3 of cartilage (deep zone) ± New collagen formation (polarized light microscopy, Picro Sirius Red stain) Cells: death, regeneration (clusters), hypertrophy, cartilage domains adjacent to fissures
	
Grade 4: erosion	Cartilage matrix loss: delamination of superficial layer, mid layer cyst formation Excavation: matrix loss superficial layer and mid zone
	
Grade 5: denudation	Surface: sclerotic bone or reparative tissue including fibrocartilage within denuded surface. Microfracture with repair limited to bone surface
	
Grade 6: deformation	Bone remodelling (more than osteophyte formation only). Includes: microfracturewith fibrocartilaginous and osseous repair extending above the previous surface

Grade = depth progression into cartilage.

**Table 3:** OA score; semi-quantitative method

	Stage % Involvement (surface, area, volume)			
	
Grade (key feature)	Stage 1 <10%	Stage 2 10-25%	Stage 3 25-50%	Stage 4 >50%
Grade 1(surface intact)	1	2	3	4

Grade 2 (surface discontinuity)	2	4	6	8

Grade 3 (vertica fissures, clefts)	3	6	9	12

Grade 4 (erosion)	4	8	12	16

Grade 5(denudation)	5	10	15	20

Grade 6(deformation)	6	12	18	24

Score = grade × stage.

### Statistical analysis

After analysis of variance, the least significant differences were used for *post hoc *testing (Scheffé's method). Average OA scores were compared and values of P < 0.05 were considered significant.

## List of abbreviations

OA: osteoarthritis; HA: hyaluronic acid; NSAIDs: nonsteroidal anti-inflammatory drugs; BSE: bovine spongiform encephalopathy; ACL-T: anterior cruciate ligament transaction; EDTA: ethylene diaminetetraacetic acid; SAF: self-assembled film.

## Competing interests

The authors declare that they have no competing interests.

## Authors' contributions

NO, MS and MK performed the research. NO and MS analyzed the data. NO took charge of the statistical analyses. MS, KU and JM wrote the manuscript. KU, TB, KT, MU and KK extracted the qniumucin from jellyfish. All authors have read and approved the final manuscript.
